# Totally Endoscopic Robotic Repair of Partial Atrioventricular Septal Defect

**DOI:** 10.1016/j.jaccas.2026.110004

**Published:** 2026-08-25

**Authors:** Natasha Mazinani, Makoto Hashimoto, Tom C. Nguyen

**Affiliations:** a Florida International University, Herbert Wertheim College of Medicine, Miami, Florida, USA; b Department of Cardiovascular Surgery, Sapporo Cardiovascular Clinic, Sapporo, Hokkaido, Japan; c Department of CardioThoracic Surgery, Miami Cardiac & Vascular Institute, Baptist Health South Florida, Miami, Florida, USA

**Keywords:** congenital heart defect, mitral valve, robotic surgery

## Abstract

**Objective:**

To demonstrate the technique of totally endoscopic robotic repair of partial atrioventricular septal defect with complex mitral valve reconstruction in an adult patient.

**Key Steps:**

Totally endoscopic robotic approach with peripheral cannulation and bicaval control. Bovine pericardial patch closure of the primum atrial septal defect. Left atriotomy for optimal mitral valve exposure. Leaflet shaving, cleft closure, autologous pericardial augmentation, and flexible annuloplasty.

**Potential Pitfalls:**

Inadequate exposure may compromise assessment of the cleft and leaflet pathology. Careful leaflet remodeling, preservation of leaflet mobility, and avoidance of excessive tension during cleft closure are essential to prevent residual regurgitation or mitral stenosis.

**Take-Home Messages:**

Totally endoscopic robotic repair enables safe and reproducible treatment of partial atrioventricular septal defect with complex mitral valve reconstruction in selected adults. Enhanced visualization and instrument dexterity facilitate precise intracardiac repair while avoiding median sternotomy.

## Visual Summary

**Figure:**
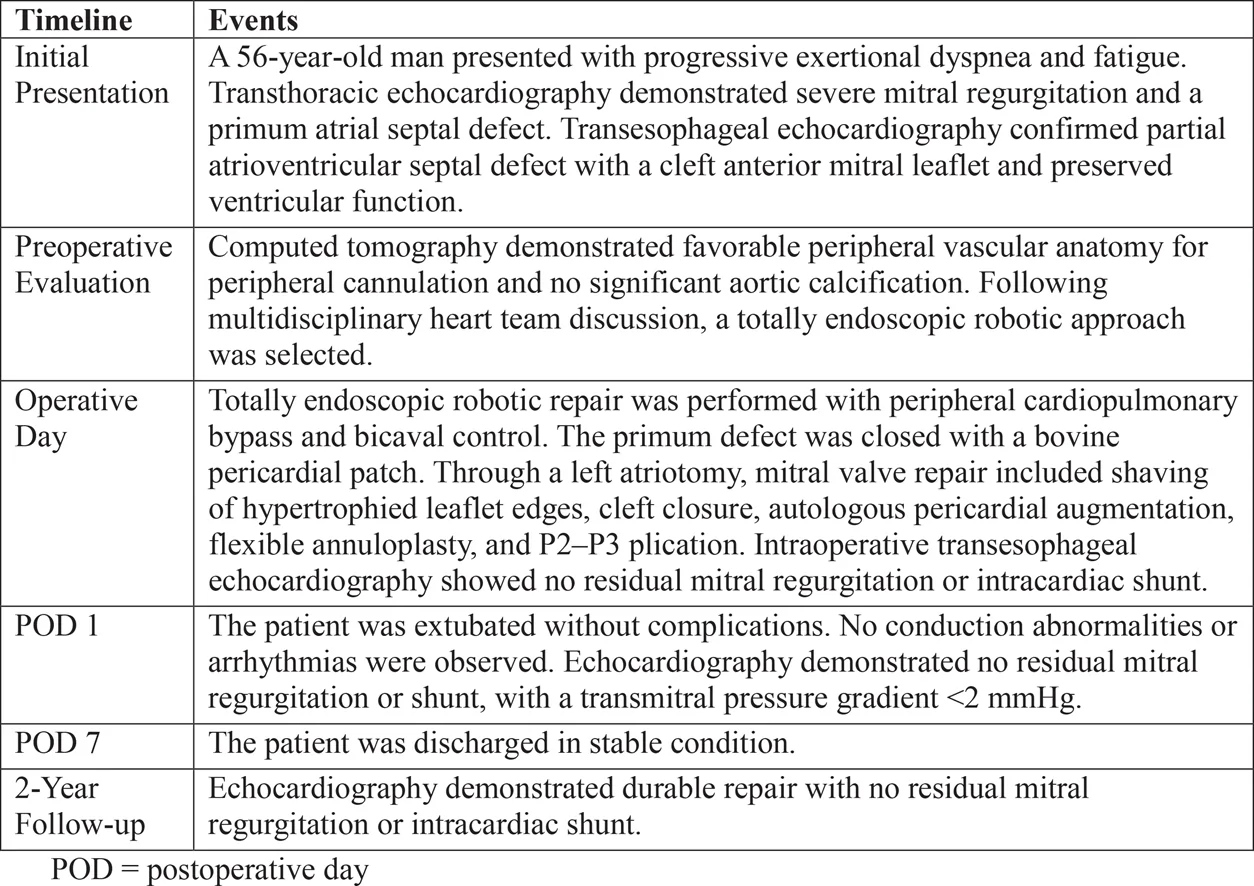

Partial atrioventricular septal defect (AVSD) is a congenital cardiac anomaly characterized by a primum atrial septal defect and a cleft left atrioventricular valve. Although the defect is usually repaired during childhood, some patients remain undiagnosed until adulthood and present with progressive mitral regurgitation, right heart volume overload, and heart failure symptoms.^1^ Surgical correction has traditionally been performed through median sternotomy.^2^^,^^3^ However, advances in robotic cardiac surgery have expanded minimally invasive options for selected adult patients requiring intracardiac repair. Although robotic repair of partial and intermediate AVSDs has previously been reported,^4^^,^^5^ detailed technical descriptions remain limited. In this report, accompanied by a comprehensive operative video, we present a totally endoscopic robotic repair of partial AVSD with severe mitral regurgitation and emphasize key technical considerations that may facilitate the safe and reproducible application of this approach in adult patients.

## Case Presentation

A 56-year-old man presented with progressive exertional dyspnea and fatigue. Transthoracic echocardiography demonstrated severe mitral regurgitation and a primum atrial septal defect. Transesophageal echocardiography confirmed partial AVSD (Figure 1) with a cleft anterior mitral leaflet (Video 1) and preserved ventricular function. Computed tomography demonstrated favorable peripheral vascular anatomy for peripheral cannulation and no significant aortic calcification. Given the patient's anatomy and preference for minimally invasive surgery, a totally endoscopic robotic approach was selected by the multidisciplinary heart team.

**Figure 1:**
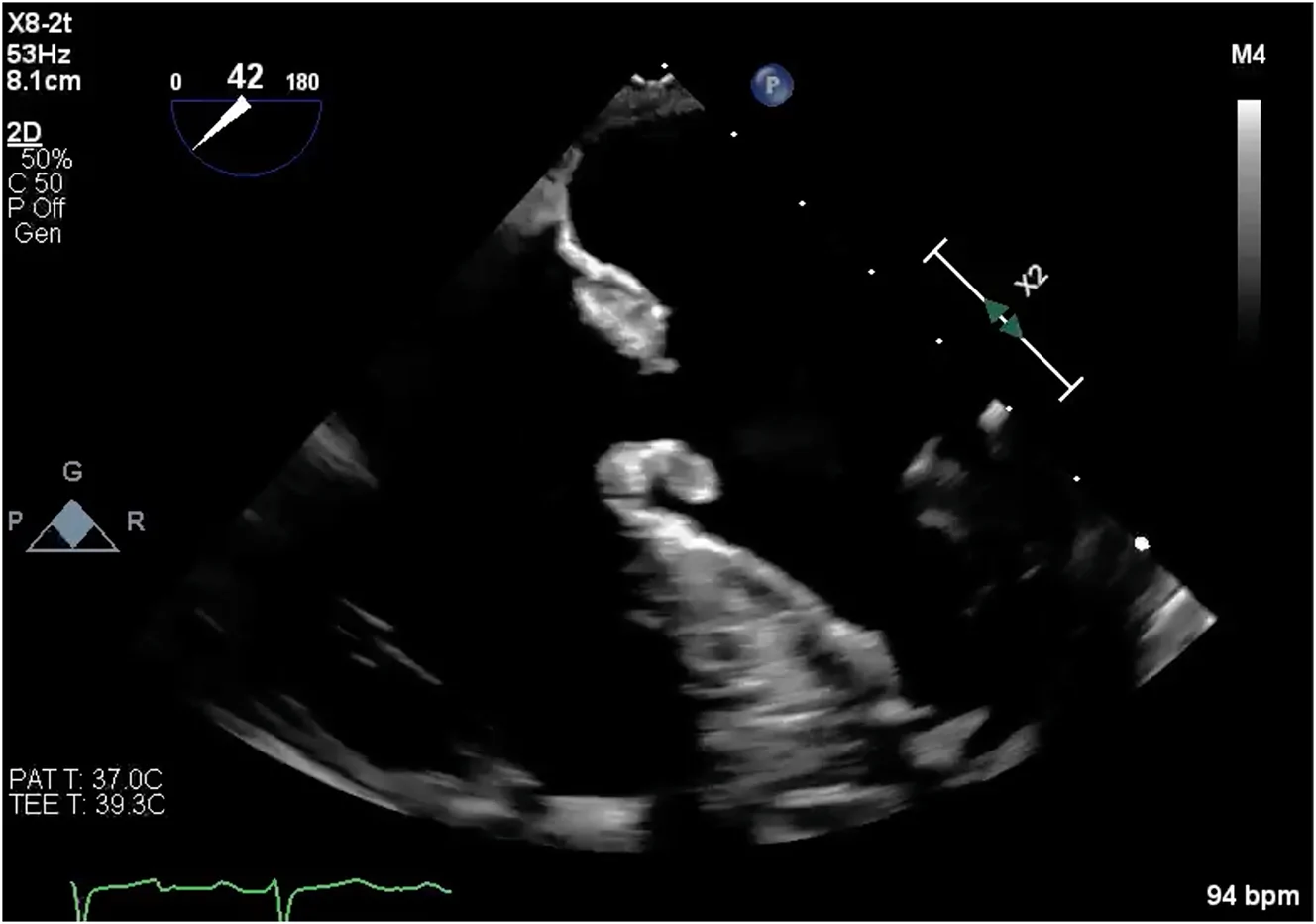
Preoperative Transesophageal Echocardiography Showing Atrio-Septal Defect

## Procedural Steps

Surgery was performed using a totally endoscopic robotic approach with the da Vinci Xi Surgical System (Intuitive Inc). Cardiopulmonary bypass was established via right femoral arterial and right femoral and jugular venous cannulation. A 3-cm working thoracotomy was created in the fourth intercostal space, and robotic ports were inserted in the third, fourth, and sixth intercostal spaces (Figure 2). Following aortic cross-clamping and bicaval control, the right atrium was opened. The primum atrial septal defect was closed using a bovine pericardial patch (Video 2). Through a left atriotomy, the cleft of the anterior mitral leaflet was identified. After shaving the hypertrophied leaflet edges, the cleft was closed with a running 5-0 polypropylene suture (Video 3). The enhanced dexterity of the robotic instruments and high-definition three-dimensional visualization facilitated precise leaflet remodeling and intracardiac manipulation. An autologous pericardial patch was added along the closure line to increase leaflet flexibility and coaptation volume. Flexible annuloplasty band implantation and P2 and P3 plication were subsequently performed (Video 4). We elected to perform annuloplasty with a ring because long-standing mitral regurgitation was expected to have resulted in annular dilatation and flattening. Saline testing demonstrated excellent valve coaptation without residual leakage. Both atria were closed simultaneously with a running 3-0 polypropylene suture, and the aorta was declamped. Transesophageal echocardiography showed no residual mitral regurgitation and no residual shunt (Videos 5 and 6). Cardiopulmonary bypass was weaned off smoothly.

**Figure 2:**
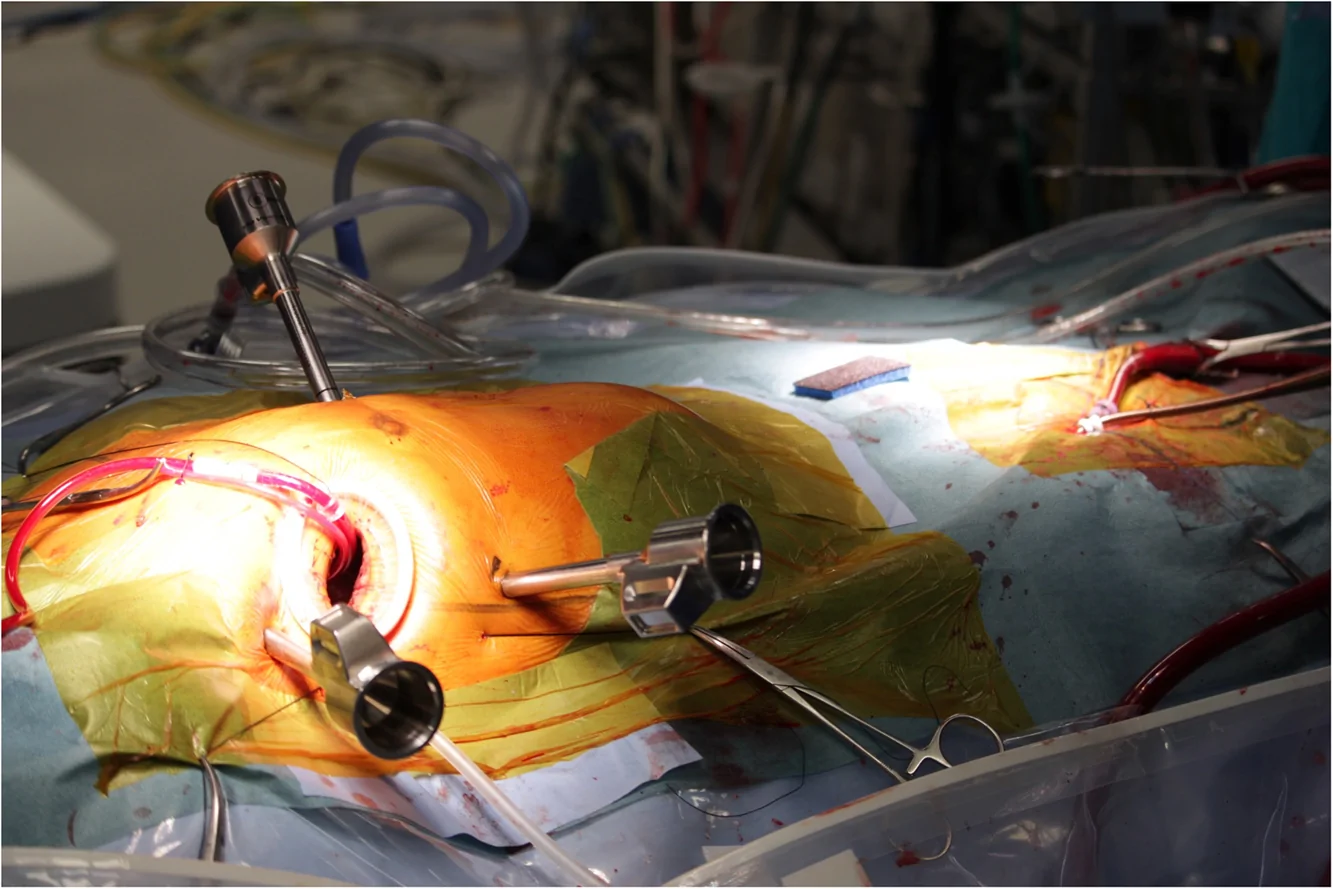
Port Placement

## Postoperative Course

The patient was extubated the morning after surgery (postoperative day 1) without complications. No conduction abnormalities or arrhythmias were observed. Echocardiography showed no residual mitral regurgitation or residual intracardiac shunt, and the transmitral pressure gradient was <2 mm Hg. The patient was discharged on postoperative day 7 in stable condition. Two-year follow-up echocardiography demonstrated no residual mitral regurgitation or intracardiac shunt.

## Discussion

Robotic cardiac surgery has become increasingly used for mitral valve procedures; however, reports describing robotic repair of congenital partial AVSD remain limited.^4^^,^^5^ In selected adult patients, robotic surgery offers several advantages, including enhanced visualization, precise intracardiac manipulation, reduced surgical trauma, and avoidance of median sternotomy.

In the present case, we anticipated that a complex mitral valve repair might be required; we elected to approach the mitral valve through a left atriotomy, and robotic visualization enabled accurate cleft assessment and repair. Furthermore, robotic instrument dexterity facilitated precise shaving and remodeling of hypertrophied leaflet tissue. Augmentation with autologous pericardium improved leaflet flexibility and coaptation without postoperative stenosis or residual regurgitation, which may contribute to improved valve durability in patients with cleft leaflet pathology. Nevertheless, the long-term outcomes of leaflet shaving and autologous pericardial augmentation remain uncertain, and continued clinical and echocardiographic follow-up is necessary to evaluate the durability of this repair strategy.

This case highlights the expanding role of robotic surgery in adult congenital intracardiac repair and demonstrates that complex valve reconstruction can be safely accomplished through a totally endoscopic approach in carefully selected patients.

## Potential Pitfalls

Adequate exposure of the mitral valve is essential for precise assessment of the cleft and leaflet pathology. Excessive leaflet shaving or tension during cleft closure should be avoided to preserve leaflet mobility and prevent residual regurgitation or mitral stenosis. During patch closure of the primum atrial septal defect, careful suture placement is also important to avoid injury to the conduction system.

## Conclusions

Totally endoscopic robotic repair of partial AVSD is feasible and safe in selected adult patients. Robotic technology may expand minimally invasive treatment options for adult congenital intracardiac pathology while providing excellent visualization and favorable early clinical outcomes.

### IRB Approval

Institutional Review Board approval was obtained.

**Take-Home Messages**

• Totally endoscopic robotic repair of partial atrioventricular septal defect is feasible and safe in carefully selected adult patients, providing excellent visualization and avoiding median sternotomy.
• High-definition robotic visualization and enhanced instrument dexterity facilitate precise cleft repair and leaflet remodeling, whereas autologous pericardial augmentation may improve leaflet coaptation and valve durability.

## Funding Support and Author Disclosures

Dr Hashimoto is a consultant for Intuitive Surgical. All other authors have reported that they have no relationships relevant to the contents of this paper to disclose.

## Appendix

Video 1 Preoperative Transesophageal Echocardiography Three-dimensional color images of mitral valve from left atrial side (left) and left ventricular side (right).

Video 2 Operative Technique; Opening Pericardium to Repair the Atrio Septal Defect

Video 3 Operative Technique; Mitral Leaflet Shaving, Autologous Pericardial Patch Augmentation

Video 4 Operative Technique; Mitral Annuloplasty, Saline Test, and Closure of Atriums

Video 5 Postoperative Transesophageal Echocardiography Three-dimensional images of the repaired mitral valve.

Video 6 Postoperative Transesophageal Echocardiography Color Doppler images of the repaired mitral valve with no residual regurgitation.
